# Admission and 24-hour heart rates and in-hospital outcomes in STEMI undergoing primary PCI

**DOI:** 10.1186/s12872-026-06027-w

**Published:** 2026-05-25

**Authors:** Yuke Cui, Zhihong Xu, Xuezheng Qu, Tianqi Zhu, Shuo Feng, Hui Han, Ruiyan Zhang, Xiaoxiang Yan, Weiwei Quan

**Affiliations:** 1 Department of Cardiovascular Medicine, Ruijin Hospital Shanghai Jiao Tong University School of Medicine, 197 Ruijin 2nd Road, Shanghai, 200025 PR China; 2 Department of Geriatric Medicine, Ruijin Hospital, Shanghai Jiao Tong University School of Medicine, 197 Ruijin 2nd Road, Shanghai, China

**Keywords:** Heart rate, Adverse in-hospital CV events, ST-segment elevation acute myocardial infarction (STEMI), Primary percutaneous coronary intervention (PPCI)

## Abstract

**Background:**

Heart rate (HR) plays a central role in cardiac physiology and has been closely associated with major cardiovascular (CV) outcomes, including heart failure, ischemic heart disease, and cardiovascular mortality. However, whether HR can serve as an early predictor of in-hospital outcomes among patients with ST-segment elevation myocardial infarction (STEMI) undergoing primary percutaneous coronary intervention (PPCI) remains unclear. This study aimed to evaluate the associations between heart rate at first medical contact (FHR), at hospital admission (AHR), and at 24 h post-admission (24 h) with adverse in-hospital outcomes.

**Methods:**

This retrospective single-center observational study included 309 consecutive patients with STEMI who underwent PPCI between September 2018 and December 2020 at Ruijin Hospital, Shanghai Jiao Tong University School of Medicine. Associations between the three HR measurements and in-hospital adverse cardiovascular events were analyzed using binary logistic regression models. Both crude models and models adjusted for conventional risk factors, biomarkers, and treatment variables were assessed.

**Results:**

Both AHR and 24 h were positively associated with in-hospital CV events, predominantly acute heart failure (*P* < 0.05), which were predominantly driven by acute heart failure. After adjusting for potential confounders, the odds of having in-hospital CV events were significantly enhanced by an increase in AHR and 24 h (odds ratio [OR]: 1.033, 95% confidence intervals [CI]: 1.008–1.060; OR: 1.037, 95% CI: 1.003–1.071, respectively). Additionally, every 5 bpm increase in AHR and 24 h was associated with an 18.6% and a 19.8% increased risk of in-hospital CV events, respectively. In contrast, FHR showed limited predictive value for such events.

**Conclusion:**

Among STEMI patients undergoing PPCI, elevated admission and 24-hour heart rates are significantly associated with adverse in-hospital cardiovascular outcomes. Admission heart rate, in particular, may serve as a simple and reliable prognostic indicator for identifying patients at increased short-term risk.

## Introduction

Heart rate (HR) can be easily and noninvasively obtained in every patient admitted with acute myocardial infarction (AMI). AMI is frequently accompanied by autonomic nervous system dysfunction, leading to marked variability in HR. An elevated HR often reflects excessive sympathetic activation and impaired vagal modulation in the early phase of AMI [1, 2] .

Previous studies have consistently demonstrated that an elevated resting HR (RHR) is associated with increased mortality risk both in the general population and among patients with cardiovascular diseases (CVDs) [3, 4]. In particular, increased HR on hospital admission has been identified as a strong predictor of higher in-hospital mortality and adverse outcomes in patients with ST-segment elevation myocardial infarction (STEMI) [5]. Furthermore, Grander et al. reported that HR measured 24 h before intensive care unit (ICU) discharge was independently associated with both in-hospital and post-discharge mortality [6]. Similarly, an elevated HR at discharge has been linked to increased long-term mortality in patients with AMI and heart failure (HF) [7].

More recently, mean HR (MHR) derived from ambulatory ECG monitoring was shown to be an independent prognostic factor for long-term all-cause mortality in patients with STEMI, with superior predictive value compared with admission or discharge HR [8]. In patients with acute myocardial infarction undergoing primary PCI, Parodi et al. also reported that elevated heart rate was independently associated with worse prognosis, further supporting the clinical relevance of early HR assessment [9]. Nevertheless, the prognostic significance of the first medical contact HR (FHR), admission HR (AHR), and the first 24-hour HR (24 h) has not been systematically investigated or compared in patients with very early-stage STEMI undergoing primary percutaneous coronary intervention (PPCI). Therefore, the present study aimed to evaluate whether elevations in FHR, AHR, and 24 h are associated with adverse in-hospital cardiovascular (CV) outcomes in patients with STEMI treated with PPCI.

## Methods

### Study population

We conducted a retrospective single-center observational study of consecutive patients with STEMI undergoing PPCI who were admitted to the Cardiac Intensive Care Unit (CICU) between September 2018 and December 2020 at Ruijin Hospital, Shanghai Jiao Tong University School of Medicine. Clinical data were retrospectively extracted from the electronic medical record system. Consecutive patients with STEMI who underwent emergency PCI were eligible for inclusion. STEMI was diagnosed according to the American Heart Association guidelines [10]. Patients who were pregnant, lactating, or younger than 18 years were excluded. Additional exclusion criteria included cardiogenic shock, severe physical disability, malignancy, autoimmune disease, or other serious systemic illnesses. Patients without analyzable sinus-rhythm heart rate measurements at the predefined assessment time points were also excluded, including those with atrial fibrillation or other rhythm disturbances that precluded reliable HR assessment. Furthermore, patients with missing key HR measurements (FHR, AHR, or 24 h) or insufficient clinical data for the predefined analyses were excluded from the final analytic cohort. The study was approved by the Institutional Review Board of Ruijin Hospital, Shanghai Jiao Tong University School of Medicine. All procedures involving human participants and human data were performed in accordance with relevant institutional requirements and the Declaration of Helsinki. Written informed consent was obtained from all patients for the use of their records for research purposes. (Clinical trial number: Not applicable)

### Patients characteristics and data acquisition

Patients’ data were acquired from the Electronic Medical Record (EMR). Baseline characteristics, including demographic data, risk factors, medical history, daily medications, laboratory tests and in-hospital events were recorded by trained medical record extractors using a standardized data collection form.

### Heart rate measurements

FHR, AHR, and 24 h were defined as the first sinus-rhythm heart rate recorded at initial medical contact, the first resting sinus-rhythm heart rate recorded after hospital admission, and the average sinus-rhythm heart rate during the first 24 h of continuous ECG monitoring in the CICU following PPCI, respectively. Initial medical contact referred to the first clinical evaluation by a healthcare provider, either in the ambulance or emergency department. HR measurements were obtained using a 12-lead resting ECG and continuous bedside ECG monitoring with the Mindray monitoring system. A resting ECG was defined as HR measured after a 5-minute period of rest in a quiet environment.

### Endpoints

The main in-hospital adverse endpoints were acute heart failure (AHF), stroke, cardiovascular (CV) death and non-cardiac death. AHF was defined based on documented clinical symptoms (e.g., breathlessness, ankle swelling and fatigue) and/or signs of heart failure during hospitalization, including dyspnea, pulmonary congestion, elevated jugular venous pressure, peripheral edema, or the requirement for heart failure-directed therapy, supported by echocardiographic findings when available [11]. Stroke was defined as the presence of a new neurologic deficit thought to be vascular in origin with signs or symptoms lasting more than 24 h. CV death was defined as death caused by myocardial infarction, deteriorative heart failure, sudden cardiac death or death caused by malignant arrhythmia and fatal valve disease. Non-cardiac death was defined as mortality that could not be explained by cardiogenic factors, including cerebral, vascular or other non-cardiac factors such as respiratory failure, infections, renal failure, liver failure and severe major bleeding.

### Statistical analysis

As this was a retrospective exploratory analysis and no prespecified imputation strategy was available, a complete-case analysis was performed. Data were analyzed with SPSS software version 20.0 (IBM Corporation, Armonk, NY, USA). All the analyses were two-sided. *P* values < 0.05 were considered statistically significant. Continuous variables were summarized as mean ± standard deviation (SD) or median (quartile range), and compared using independent student’s t-test, one-way ANOVA or Kruskal-Wallis H-test where appropriate. Categorical variables were expressed as percentages and frequencies, and compared using the chi-squared test or Fisher exact test.

Tertile categorization was mainly used for descriptive baseline comparisons, whereas regression analyses primarily evaluated HR as continuous variables, with the first and third tertiles representing the lowest and highest tertile, respectively. Binary logistic regression was used to evaluate the odds ratios (ORs) for the associations between the adverse in-hospital CV events and the three HR measurements with the lowest HR tertile as a reference. Covariates were selected based on clinical relevance, prior literature, and data availability and were entered sequentially into multivariable models, a *P*-value of ≤ 0.05 was the criterion to enter the model. Adjustment for the relevant covariates was performed stepwise in five models. Model 1: adjustment for sex and age, Model 2: additional adjustment for Body Mass Index (BMI), Diastolic Blood Pressure (DBP), smoking status, hypertension, diabetes and CVD, Model 3: further adjustment for high-sensitivity C-Reactive Protein (hs-CRP) and N-terminal pro brain natriuretic peptide (NT-proBNP), Model 4: additional adjustment for fasting plasma glucose (FPG), postprandial glucose(PPG), microalbuminuria (ALBu) and estimated glomerular filtration rate (eGFR), Model 5: additional adjustment for the usage of b-blockers, Model 6: further adjustment for other related medications [angiotensin-converting enzyme-inhibitors (ACEI)/angiotensin II receptor blockers (ARB), statins, spironolactone and nitrates]. The adjusted ORs with their respective 95% confidence intervals (CIs) for each group were calculated. *P*-values < 0.05 were considered statistically significant.

## Results

### Characteristics of the study population

**Fig. 1:**
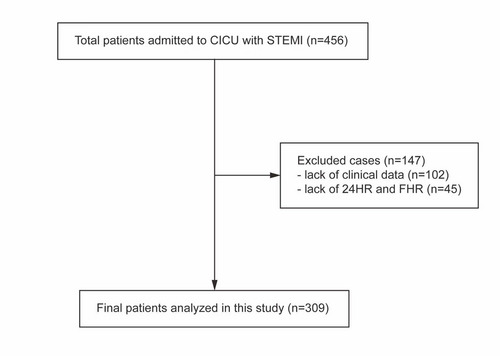
Flowchart illustrating the sample selection for the present analysis. STEMI: ST-segment elevation myocardial infarction; 24 h: 24-hour heart rate ; FHR: first medical contact heart rate

A total of 309 patients with STEMI undergoing PPCI were included in the final analysis (Fig. 1). The demographic data of the study population were divided into three groups according to the tertiles of all the three HR measurements for further analysis (Table 1). The mean age was 65.18 ± 12.48 years, and most of them were male patients (*N* = 240, 77.7%). The prevalence of all the in-hospital CV events was 32.0%. The baseline admission HR was higher in patients with in-hospital CV events (*P* < 0.001). Patients with in-hospital CV events had higher levels of hs-CRP, NT-proBNP and cTnI and lower LVEF than those without events (*P* < 0.05).

**Table 1:** Baseline characteristics of study population

		All patients	In-hospital events	No in-hospital events	*P*
*N*		309	99	210	
Demographic and risk factors					
	age	65.18(12.48)	70.89(11.34)	62.51(12.11)	< 0.001
	male	240(77.7%)	75(75.8%)	165(78.6%)	0.579
	BMI	24.27(3.52)	23.79(3.74)	24.50(3.40)	0.098
	baseline SBP	124.48(20.91)	123.96(21.40)	124.72(20.73)	0.765
	baseline DBP	75.99(13.59)	75.34(14.06)	76.29(13.39)	0.574
	baseline HR	84.36(15.67)	89.91(16.70)	81.75(14.48)	< 0.001
	smoking status	157(50.8%)	48(48.5%)	109(51.9%)	0.632
Medical history					
	hypertension	183(59.2%)	62(62.6%)	121(57.6%)	0.403
	diabetes	87(28.2%)	29(29.3%)	58(27.6%)	0.760
	CVD	84(27.2%)	36(36.4%)	48(27.6%)	0.013
Clinical presentation					
	hs-CRP	16.60(8.10)	51.97(13.91-142.94)	12.84(7.30-41.53)	< 0.001
	NT-proBNP	1243.00(419.70–3311.00)	3259.50(1425.25-7907.25)	754.90(268.50–1945.00)	< 0.001
	cTNI	37.75(12.92-105.95)	48.34(14.10-165.67)	33.50(11.34–81.25)	0.011
	CKMB	158.80(49.80–304.00)	186.00(39.30-361.80)	149.05(57.90–303.00)	0.387
	LVEF	57.11(8.17)	52.26(8.94)	59.35(6.72)	< 0.001
	FPG	6.80(2.80)	7.23(2.98)	6.63(2.71)	0.098
	PPG	10.27(4.09)	10.79(4.39)	10.06(3.96)	0.171
	HbA1c	6.39(1.43)	6.53(1.55)	6.33(1.37)	0.252
	eGFR	76.88(23.64)	64.80(25.72)	82.58(20.29)	< 0.001
	AlbU	2.11(1.09–6.94)	4.29(1.38-13.00)	1.73(1.07–4.78)	< 0.001
Culprit vessel					
	LAD	161(52.1%)	50(50.5%)	111(52.9%)	0.764
	LCX	34(11.0%)	9(9.1%)	25(11.9%)	
	RCA	101(32.7%)	35(35.4%)	66(31.4%)	
	Others	13(4.2%)	5(5.1%)	8(3.8%)	
	multi-vessel disease	243(78.6%)	81(81.8%)	162(77.5%)	0.387
medications					
	DAPT	309(100%)	99(100%)	210(100%)	NA
	beta-blocker	273(88.3%)	85(85.9%)	188(89.5%)	0.349
	ACEI/ARB	230(74.4%)	69(69.7%)	161(76.7%)	0.190
	statins	295(95.5%)	90(90.9%)	205(97.6%)	0.191
	spironolactone	29(9.4%)	20(20.2%)	9(4.3%)	< 0.001
	diuretics	35(11.3%)	24(24.2%)	11(5.2%)	< 0.001
	nitrates	102(33.0%)	46(46.5%)	56(26.7%)	0.001
Hospital events		99(32.0%)	99(100%)	NA	NA
	AHF	91(29.4%)	91(91.9%)	NA	NA
	stroke	10(3.2%)	10(10.1%)	NA	NA
	cardiac death	14(4.5%)	14(14.1%)	NA	NA
	non cardiac death	4(1.3%)	4(4.0%)	NA	NA

Continuous variables are summarized as the mean (SD) or median (quartile range) where appropriate, and were compared using student t-test and Mann-Whitney U test. Categorical variables are expressed as percentages and frequencies of the cohort and were compared using the chi-squared test or Fisher exact test

*BMI* body mass index, *SBP* Systolic blood pressure, *DBP *diastolic blood pressure, *CVD* cardiovascular disease, *hs-CRP* high sensitivity C Reactive Protein, *NT-proBNP* N-terminal pro brain natriuretic peptide, *cTNI* cardiac troponin I, *CK-MB* creatine kinase-MB, *LVEF* left ventricular ejection fraction, *Hb *hemoglobin, *FPG* fasting plasma glucose, *PPG* postprandial glucose, *HbA1C* hemoglobin A1C, *TC* serum total cholesterol, *TG* Triglyceride, *HDL* high-density lipoprotein, *LDL* low-density lipoprotein, *APOB* apolipoprotein B, *LPA* lipoprotein A, *eGFR* estimated glomerular filtration rate, *ALBu* microalbuminuria, *LAD* left anterior descending, *LCX* left circumflex artery, *RCA* right coronary artery, *DAPT* dual-antiplatelet-therapy, *ACEI* angiotensin-converting enzyme inhibitor, *ARB* angiotensin receptor inhibitor, *AHF* acute heart failure

Tables 2, 3 and 4 demonstrate the characteristics of the study population according to the tertiles of AHR, 24 h and FHR, respectively. In the highest tertile of HR for AHR and 24 h, the patients had a greater incidence of in-hospital CV events (*P* < 0.01). Higher levels of AHR and 24 h were associated with higher baseline DBP, poorer cardiac and renal function and more serious inflammation and myocardial injury status (*P* < 0.05). The level of AHR positively correlated with DBP and specific biomarkers, involving hs-CRP, NT-proBNP, cTnI, CK-MB and ALBU (all *P* < 0.05, Table 2). Furthermore, FPG and PPG were higher in the group presenting with higher 24 h (all *P* < 0.05, Table 3). The left anterior descending artery (LAD) was most often the culprit vessel on coronary angiography in patients with higher 24 h and FHR (Tables 3 and 4). Compared with the group presenting with lower 24 h, most patients with higher 24 h received b-blocker and diuretics (all *P* < 0.05, Table 3). Patients with higher FHR seemed to show higher levels of NT-proBNP, CK-MB and eGFR (all *P* < 0.05, Table 4). Eighteen patients experienced more than one event during hospitalization; however, each patient was counted only once in the composite endpoint analysis.

**Table 2:** Baseline characteristics according to AHR tertiles

		AHR			
		T1(< 76.00)	T2(76.00–90.00)	T3(≥ 90.00)	*P*
N		103	105	101	
Demographic and risk factors					
	age	63.72(12.37)	65.22(12.19)	66.62(12.82)	0.251
	male	80(77.7%)	80(76.2%)	80(79.2%)	0.874
	BMI	24.43(3.21)	24.56(4.03)	23.81(3.22)	0.263
	baseline SBP	124.22(21.37)	124.19(20.59)	125.04(20.98)	0.948
	baseline DBP	73.45(13.84)	76.20(13.51)	78.33(13.11) *	0.037
	baseline HR	67.58(6.59)	83.74(3.91) *	102.12(9.34) *†	< 0.001
	smoking status	60(58.3%)	48(45.7%)	49(48.5%)	0.174
Medical history					
	hypertension	61(59.2%)	62(59.0%)	60(59.4%)	0.999
	diabetes	27(26.2%)	25(23.8%)	35(34.7%)	0.194
	CVD	29(28.2%)	29(27.6%)	26(25.7%)	0.921
Clinical presentation					
	hs-CRP	13.75(7.31–52.69)	15.14(7.66–55.81)	32.36(10.48-119.74) *†	0.004
	NT-proBNP	947.20(270.23-1969.50)	1039.00(433.38-3114.25)	1981.00(587.65–5275.00) *†	0.001
	cTnI	30.04(8.50–81.00)	43.06(17.09-109.76)	38.57(13.66-165.77)	0.039
	CK-MB	121.00(30.90-262.10)	182.40(62.60-331.75)	172.50(52.05-428.45)	0.040
	LVEF	59.50(7.45)	57.33(7.38)	54.40(8.89) *†	< 0.001
	FPG	6.64(3.24)	6.68(2.32)	7.12(2.77)	0.429
	PPG	10.32(4.41)	10.09(3.65)	10.42(4.24)	0.851
	HbA1c	6.39(1.41)	6.26(1.14)	6.53(1.70)	0.413
	eGFR	79.38(22.47)	75.70(23.74)	75.56(24.71)	0.424
	AlbU	1.73(1.08–4.51)	1.80(1.09–7.04)	3.60(1.11–10.95) *	0.030
Culprit vessel					
	LAD	54(52.4%)	53(50.5%)	54(53.5%)	0.991
	LCX	11(10.7%)	11(10.5%)	12(11.9%)	
	RCA	33(32.0%)	36(34.3%)	32(31.7%)	
	Others	5(4.9%)	5(4.8%)	3(3.0%)	
	multi-vessel disease	78(75.7%)	81(77.1%)	84(83.2%)	0.436
Medications					
	DAPT	103(100%)	105(100%)	101(100%)	NA
	beta-blocker	86(83.5%)	97(92.4%)	90(89.1%)	0.131
	ACEI/ARB	78(75.7%)	79(75.2%)	73(72.3%)	0.830
	statins	99(95.1%)	101(96.2%)	95(94.1%)	0.743
	spironolactone	10(9.7%)	6(5.7%)	13(12.9%)	0.210
	diuretics	10(9.7%)	8(7.6%)	17(16.8%)	0.093
	nitrates	34(33.0%)	37(35.2)	31(30.7%)	0.786
Hospital events		22(21.4%)	32(30.5%)	45(44.6%) *	0.002
	AHF	20(19.4%)	28(26.7%)	43(42.6%) *†	0.001
	stroke	2(1.9%)	2(1.9%)	6(5.9%)	0.189
	cardiac death	1(1.0%)	3(2.9%)	10(9.9%) *	0.009
	non cardiac death	0(0%)	1(1.0%)	3(3.0%)	0.129

Continuous variables are summarized as the mean (SD) or median (quartile range) where appropriate, and were compared using one-way ANOVA analysis and Kruskal-Wallis test. Categorical variables are expressed as percentages and frequencies of the cohort and were compared using the chi-squared test or Fisher exact test

*AHR* admission heart rate, *BMI* body mass index, *SBP* systolic blood pressure, *DBP* diastolic blood pressure, *CVD* cardiovascular disease, *hs-CRP* high sensitivity high C Reactive Protein, *NT-proBNP* N-terminal pro brain natriuretic peptide, *cTnI* cardiac troponin I, *CK-MB* creative kinase isoenzyme, *LVEF* left ventricular ejection fraction, *Hb* hemoglobin, *FPG* fasting plasma glucose, *PPG* postprandial glucose, *HbA1C* hemoglobin A1C, *TC* serum total cholesterol, *TG* triglyceride, *HDL* high-density lipoprotein, *LDL* low-density lipoprotein, *APOB* apolipoprotein B, *LPA* lipoprotein A, *eGFR* estimated glomerular filtration rate, *ALBu* microalbuminuria, *LAD* left anterior descending, *LCX* left circumflex artery, *RCA* right coronary artery, *DAPT* dual-antiplatelet-therapy, *ACEI* angiotensin-converting enzyme inhibitor, *ARB* angiotensin receptor inhibitor, *AHF* acute heart failure

*P < 0.05, compared with lowest tertile group; †*P* < 0.05, compared with the middle tertile group

**Table 3:** Baseline characteristics according to admission 24-hour HR tertiles

		admission 24-hour HR			
		T1(< 70.71)	T2(70.71–80.29)	T3(≥ 80.29)	*P*
N		102	104	103	
Demographic and risk factors					
	age	64.74(11.43)	63.58(12.61)	67.22(13.15)	0.100
	male	85(83.3%)	78(75.0%)	77(74.8%)	0.244
	BMI	24.46(3.10)	24.55(3.90)	23.80(3.49)	0.249
	baseline SBP	123.27(21.78)	124.15(19.34)	126.00(21.66)	0.636
	baseline DBP	73.76(14.54)	75.63(11.29)	78.52(14.44)*	0.041
	baseline HR	74.15(12.49)	82.22(11.62)*	96.64(13.76)*†	< 0.001
	smoking status	55(53.9%)	54(51.9%)	48(46.6%)	0.601
Medical history					
	hypertension	66(64.7%)	59(56.7%)	58(56.3%)	0.387
	diabetes	23(22.5%)	26(25.0%)	38(36.9%)	0.050
	CVD	27(26.5%)	26(25.0%)	31(30.1%)	0.698
Clinical presentation					
	hs-CRP	11.16(6.36–33.39)	14.48(6.80-53.36)	53.90(14.84-134.52)*†	< 0.001
	NT-proBNP	711.70(254.35-1521.50)	922.50(325.00-2319.00)	3165.00(1299.00-6842.00)*†	< 0.001
	cTnI	34.84(11.87–77.47)	33.73(11.41-127.95)	44.41(13.79-154.97)	0.066
	CK-MB	137.95(60.53-267.05)	173.45(65.83-333.03)	162.60(42.80-393.60)	0.198
	LVEF	59.79(6.49)	58.15(7.46)	53.33(9.03)*†	< 0.001
	FPG	6.41(3.41)	6.63(2.04)	7.41(2.70)*	0.038
	PPG	9.65(3.91)	10.06(3.70)	11.21(4.55)*	0.025
	HbA1c	6.21(1.27)	6.40(1.39)	6.56(1.60)	0.212
	eGFR	77.02(21.32)	79.53(22.24)	74.07(26.89)	0.252
	AlbU	1.71(1.07–4.83)	1.72(1.08–5.75)	3.83(1.36-12.00)*†	0.001
Culprit vessel					
	LAD	38(37.3%)	59(56.7%)	64(62.1%)	0.004
	LCX	10(9.8%)	13(12.5%)	11(10.7%)	
	RCA	47(46.1%)	30(28.8%)	24(23.3%)	
	Others	7(6.9%)	2(1.9%)	4(3.9%)	
	multi-vessel disease	82(80.4%)	81(77.9%)	80(77.7%)	0.788
Medications					
	DAPT	102(100%)	104(100%)	103(100%)	NA
	beta-blocker	83(81.4%)	96(92.3%)	94(91.3%)	0.027
	ACEI/ARB	75(73.5%)	80(76.9%)	75(72.8%)	0.769
	statins	98(96.1%)	101(97.1%)	96(93.2%)	0.405
	spironolactone	6(5.9%)	9(8.7%)	14(13.6%)	0.159
	diuretics	8(7.8%)	8(7.7%)	19(18.4%)	0.020
	nitrates	35(34.3%)	32(30.8%)	35(34.0%)	0.836
Hospital events		22(21.6%)	24(23.1%)	53(51.5%)*†	< 0.001
	AHF	19(18.6%)	21(20.2%)	51(49.5%)*†	< 0.001
	stroke	3(2.9%)	2(1.9%)	5(4.9%)	0.485
	cardiac death	2(2.0%)	0(0%)	12(11.7%)*†	< 0.001
	non cardiac death	0(0%)	1(1.0%)	3(2.9%)	0.229

Continuous variables are summarized as the mean (SD) or median (quartile range) where appropriate, and were compared using one-way ANOVA analysis and Kruskal-Wallis test. Categorical variables are expressed as percentages and frequencies of the cohort and were compared using the chi-squared test or Fisher exact test

*BMI* body mass index, *SBP* Systolic blood pressure, *DBP* diastolic blood pressure, *CVD* cardiovascular disease, *hs-CRP* high sensitivity high C Reactive Protein, *NT-proBNP* N-terminal pro brain natriuretic peptide, *cTnI* cardiac troponin I, *CK-MB* creative kinase isoenzyme, *LVEF* left ventricular ejection fraction, *Hb* hemoglobin, *FPG* fasting plasma glucose, *HbA1C* Hemoglobin A1C, *TC* Serum total cholesterol, *TG* Triglyceride, *HDL* High-density lipoprotein, *LDL* low-density lipoprotein, *APOB* apolipoprotein B, *LPA* lipoprotein A, *eGFR* estimated glomerular filtration rate, *ALBu* microalbuminuria, *LAD* left anterior descending, *LCX* left circumflex artery, *RCA* right coronary artery, *DAPT* Dual-antiplatelet-therapy, *ACEI* angiotensin-converting enzyme inhibitor, *ARB* angiotensin receptor inhibitor, *AHF* acute heart failure

*P < 0.05, compared with lowest tertile group; †*P* < 0.05, compared with the middle tertile group

**Table 4:** Baseline characteristics according to FHR tertiles

		FHR			
		T1(< 69.00)	T2(69.00–85.00)	T3(≥ 85.00)	*P*
N		104	105	100	
Demographic and risk factors					
	age	66.32(12.86)	64.50(11.43)	64.72(13.15)	0.520
	male	76(73.1%)	82(78.1%)	82(82.0%)	0.308
	BMI	23.89(3.40)	24.19(3.08)	24.75(4.01)	0.206
	baseline SBP	120.27(19.61)	125.44(21.08)	127.85(21.50)*	0.029
	baseline DBP	73.09(14.06)	76.52(13.14)	78.41(13.15)*	0.018
	baseline HR	78.30(15.73)	85.01(15.36)*	89.99(13.70)*	< 0.001
	smoking status	55(52.9%)	56(53.3%)	46(46.0%)	0.551
Medical history					
	hypertension	61(58.7%)	62(59.0%)	60(60.0%)	0.980
	diabetes	28(26.9%)	26(24.8%)	33(33.0%)	0.399
	CVD	32(30.8%)	24(22.9%)	28(28.0%)	0.427
Clinical presentation					
	hs-CRP	15.45(7.99–44.75)	17.39(7.82–62.06)	17.96(8.50-96.86)	0.264
	NT-proBNP	897.40(300.23-2230.50)	1122.50(353.48–3356.00)	1876.00(496.80–4549.00)*	0.011
	cTnI	42.43(17.09–92.85)	43.06(12.33–142.00)	30.18(10.54–76.22)	0.166
	CK-MB	163.55(63.28–304.00)	202.00(78.55-402.65)*	117.15(37.80-254.33)	0.022
	LVEF	59.13(6.78)	56.81(8.89)	55.33(8.32)*	0.004
	FPG	6.86(3.41)	6.65(2.35)	6.91(2.56)	0.797
	PPG	10.21(4.37)	9.93(3.53)	10.70(4.34)	0.433
	HbA1c	6.30(1.38)	6.27(1.23)	6.60(1.65)	0.199
	eGFR	74.75(24.15)	77.58(22.81)	78.37(24.05)	0.515
	AlbU	2.33(1.09–5.66)	1.93(1.09–5.97)	2.48(1.09–9.41)	0.566
Culprit vessel					
	LAD	41(39.4%)	56(53.3%)	64(64.0%)*	0.003
	LCX	11(10.6%)	16(15.2%)	7(7.0%)	
	RCA	48(46.2%)	30(28.6%)*	23(23.0%)*†	
	Others	4(3.8%)	3(2.9%)	6(6%)	
	multi-vessel disease	76(73.1%)	85(81.0%)	82(82.0%)	0.202
Medications					
	DAPT	104(100%)	105(100%)	100(100%)	NA
	beta-blocker	85(81.7%)	91(86.7%)	97(97.0%)*†	0.002
	ACEI/ARB	68(66.3%)	83(79.0%)	78(78.0%)	0.067
	statins	98(94.2%)	102(97.1%)	95(95.0%)	0.586
	spironolactone	7(6.7%)	11(10.5%)	11(11.0%)	0.518
	diuretics	12(11.5%)	11(10.5%)	12(12.0%)	0.939
	nitrates	37(35.6%)	35(33.3%)	30(30.0%)	0.696
Hospital events		36(34.6%)	25(23.8%)	38(38.0%)	0.074
	AHF	34(32.7%)	24(22.9%)	33(33.0%)	0.189
	stroke	2(1.9%)	3(3.9%)	5(5.0%)	0.439
	cardiac death	2(1.9%)	4(3.8%)	8(8.0%)	0.117
	non cardiac death	2(1.9%)	2(1.9%)	0(0%)	0.552

Continuous variables are summarized as the mean (SD) or median (quartile range) where appropriate, and were compared using one-way ANOVA analysis and Kruskal-Wallis test. Categorical variables are expressed as percentages and frequencies of the cohort and were compared using the chi-squared test or Fisher exact test

*FHR* First medical contact heart rate, *BMI* body mass index, *SBP* systolic blood pressure, *DBP* diastolic blood pressure, *CVD* cardiovascular disease, *hs-CRP* high sensitivity high C Reactive Protein, *NT-proBNP* N-terminal pro brain natriuretic peptide, *cTnI* cardiac troponin I, *CK-MB* creative kinase isoenzyme, *LVEF* left ventricular ejection fraction, *Hb* hemoglobin, *FPG* fasting plasma glucose, *PPG* postprandial glucose, *HbA1C* hemoglobin A1C, *TC* serum total cholesterol, *TG* triglyceride, *HDL* high-density lipoprotein, *LDL* low-density lipoprotein, *APOB* apolipoprotein B, *LPA* lipoprotein A, *eGFR* estimated glomerular filtration rate, *ALBu* microalbuminuria, *LAD* left anterior descending, *LCX* left circumflex artery, *RCA* right coronary artery, *DAPT* dual-antiplatelet-therapy, *ACEI* angiotensin-converting enzyme inhibitor, *ARB* angiotensin receptor inhibitor, *AHF* acute heart failure

*P < 0.05, compared with lowest tertile group; †*P* < 0.05, compared with the middle tertile group

### Association of in-hospital CV events with heart rate level

Among the 99 patients (32.0%) who experienced adverse in-hospital CV events, 91 (29.4%) developed AHF, 10 (3.2%) had stroke and 18 (5.8%) died [14 (4.5%) of them were cardiac death and 4 (1.3%) were non-cardiac death] (Table 1). Furthermore, 18 patients experienced more than one event during hospitalisation. Among the 91 patients with AHF, 4 developed stroke, 13 died from in-hospital events and 3 died from non-cardiac factors. Comparing AHR and 24 h, the patients with the third tertile HR were more likely to develop adverse in-hospital CV events. Additionally, most patients with higher AHR and 24 h suffered from cardiac death compared to the group with lower HR. No significant difference in the prevalence of in -hospital CV events was observed among the FHR tertiles.

In a multivariable logistic regression, AHR and 24 h were significantly associated with adverse in-hospital CV events both in crude and conventional cardiovascular risk factors adjusted analysis (Table 5; Model 1, Model 2 and Model 3). In the crude model, both AHR and 24 h, assessed as continuous variables, were significantly associated with adverse in-hospital CV events. For every 1 bpm increase in AHR and 24 h, the odds of adverse in-hospital CV events increased by 3.5% and 5.3%, respectively (OR 1.035, 95% CI 1.018–1.052; OR 1.053, 95% CI 1.032–1.075). In contrast, FHR was not significantly associated with adverse in-hospital CV events in either the univariate or multivariable analysis. After adjusting for age and sex, this association remained statistically significant with adjusted ORs of 1.034 (95% CI 1.016–1.051) and 1.058 (95% CI 1.034–1.081), respectively. Additionally, after controlling for the confounding effects of other risk factors including BMI, DBP, smoking status, hypertension, diabetes and CVD history, there was a 3.9% and 6.4% higher probability of developing new adverse in-hospital CV events for every increment in AHR and 24 h (OR: 1.039, 95% CI: 1.020–1.058; OR 1.064, 95% CI: 1.039–1.090, respectively). After further adjustment for hs-CRP and NT-proBNP, the association was persistently significant (adjusted OR [aOR]: 1.122, 95% CI: 1.016–1.240 and aOR: 1.168, 95% CI: 1.021–1.335) (Table 5; Model 3) for every 5 bpm increase in AHR and 24 h, respectively. However, the association between 24 h and adverse in-hospital events disappeared after adjusting for more biomarkers, including FPG, PPG, ALBU and eGFR (Table 5; Model 4) (OR: 1.029, 95% CI: 0.997–1.062, *P* = 0.074). The relationship between AHR and worse in-hospital outcomes remained significant (OR: 1.026, 95% CI: 1.002–1.050, *P* = 0.035). Furthermore, the association between every 5 bpm increase in 24 h and adverse in-hospital events reappeared after adjusting for beta-blockers use (OR: 1.176, 95% CI: 1.003–1.379, *P* = 0.046) (Table 5; Model 4). After adjusting for other relevant medications (ACEI/ARB, statins, spironolactone and nitrates), AHR remained significantly associated with adverse in-hospital CV events. There was an 18.6% and a 19.8% higher probability of developing new adverse outcomes for every 5 bpm increase in AHR and 24 h, respectively after controlling for the confounding effects of full risk factors and medications (Table 5; Model 6). Nevertheless, regardless of the univariate or multivariable adjusted analysis, there was no association between adverse in-hospital CV events and FHR.

**Table 5:** Binary logistic analysis for the risk of in-hospital events

		AHR			admission 24-hour HR			FHR		
		OR	95% CI	*P*	OR	95% CI	*P*	OR	95% CI	*P*
unadjusted	continuous	1.035	1.018–1.052	< 0.001	1.053	1.032–1.075	< 0.001	0.999	0.986–1.012	0.884
	per 5 bpm	1.191	1.098–1.292	< 0.001	1.296	1.170–1.435	< 0.001	1.000	0.939–1.065	0.991
Model1	continuous	1.034	1.016–1.051	< 0.001	1.058	1.034–1.081	< 0.001	1.001	0.987–1.014	0.940
	per 5 bpm	1.183	1.086–1.288	< 0.001	1.322	1.184–1.476	< 0.001	1.006	0.942–1.075	0.850
Model2	continuous	1.039	1.020–1.058	< 0.001	1.064	1.039–1.090	< 0.001	1.002	0.988–1.015	0.820
	per 5 bpm	1.212	1.107–1.328	< 0.001	1.361	1.208–1.532	< 0.001	1.012	0.946–1.083	0.731
Model3	continuous	1.023	1.002–1.043	0.028	1.032	1.004–1.060	0.025	0.985	0.970–1.001	0.066
	per 5 bpm	1.122	1.016–1.240	0.023	1.168	1.021–1.335	0.023	0.929	0.859–1.006	0.069
Model4	continuous	1.026	1.002–1.050	0.035	1.029	0.997–1.062	0.074	0.984	0.965–1.002	0.081
	per 5 bpm	1.145	1.017–1.288	0.025	1.157	0.991–1.349	0.065	0.925	0.843–1.015	0.099
Model5	continuous	1.027	1.003–1.052	0.029	1.033	1.000-1.067	0.052	0.984	0.965–1.003	0.091
	per 5 bpm	1.152	1.022–1.299	0.021	1.176	1.003–1.379	0.046	0.926	0.843–1.018	0.111
Model6	continuous	1.033	1.008–1.060	0.011	1.037	1.003–1.071	0.032	0.985	0.966–1.004	0.128
	per 5 bpm	1.186	1.046–1.344	0.008	1.198	1.021–1.406	0.027	0.933	0.848–1.027	0.155

logistics regression was performed. Odds ratio for each increment of 1 and 5 bpm was shown, respectively. Model1 adjusted for age and sex; Model2 adjusted for terms in Model1 and BMI, DBP, smoking status, hypertension, diabetes, CVD history; Model3 adjusted for terms in Model2 and hs-CRP, NT-proBNP; Model4 adjusted for terms in Model3 and FPG, PPG, ALBU, eGFR; Model5 adjusted for terms in Model4 and usage of beta-blockers; Model6 adjusted for terms in Model5 and usage of ACEI/ARB, statins, spironolactone, nitrates

## Discussion

In current study, after adjusting for multiple confounders including sociodemographic factors, clinical biomarkers and medication usage, AHR and 24 h were significantly associated with the occurrence of in-hospital CV events, whereas FHR was not significantly associated with in-hospital events in either univariate or multivariable analysis. In addition, AHR demonstrated the most consistent association with adverse in-hospital outcomes across multivariable models. Importantly, the composite endpoint was predominantly driven by acute heart failure, whereas stroke and death events were relatively infrequent. Therefore, the observed associations should primarily be interpreted as reflecting the relationship between elevated HR and early in-hospital heart failure risk.

HR is a fundamental, vital and noninvasively measured physiological parameter. Previous epidemiological studies have shown that HR is a strong predictive value for cardiovascular morbidity and mortality, indicating that HR measurement should be an indispensable component of cardiovascular disease risk evaluation [12]. Elevated HR, which reflects sympathetic hyperactivity, impaired cardiac function, and reduced ventricular ejection fraction, has been linked to increased mortality and CV events not only in patients with cardiovascular disease (CVD) but also among those with hypertension, metabolic syndrome, and even in the elderly and otherwise healthy populations [13–19]. Previous studies have demonstrated that a higher AHR adversely affects both short- and long-term outcomes in AMI [17, 20, 21] and that elevated 24 h is associated with long-term adverse outcomes following AMI [8, 22]. However, limited data exist regarding the prognostic significance of FHR and 24 h for in-hospital outcomes. In our cohort of STEMI patients undergoing PPCI, both AHR and 24 h were significantly associated with in-hospital adverse CV events. However, this relationship of 24 h was less consistent after extensive adjustment and should therefore be interpreted cautiously. Notably, the composite endpoint was predominantly driven by acute heart failure, which accounted for the majority of in-hospital events in this cohort.

Our findings are consistent with prior reports showing that elevated AHR predicts in-hospital complications and mortality in patients with CVD and acute ischemic stroke [5, 8, 23]. Following AMI, autonomic imbalance characterized by sympathetic predominance and parasympathetic withdrawal occurs almost immediately [24]. Because AHR reflects the patient’s autonomic status at the time of admission, it provides valuable early prognostic information. Indeed, both in-hospital and post-discharge mortality increase linearly with AHR [21, 25], supporting its relevance as a short-term prognostic indicator.

The average 24 h, measured through continuous monitoring after PCI, may provide a more integrated—albeit delayed—representation of changes in cardiac and autonomic function. Elevated average 24 h has been identified as an independent predictor of new coronary events [26]. Because 24 h was measured after hospital admission and PPCI, it may partly reflect evolving clinical deterioration, treatment response, or early complications such as heart failure rather than functioning solely as an independent baseline prognostic marker. Therefore, reverse causation and time-dependent bias cannot be excluded. In our study, 24 h was initially associated with in-hospital CV events, but this relationship disappeared after adjustment for biomarkers such as FPG, PPG, ALBu, and eGFR. This suggests that 24 h may reflect autonomic dysfunction in conjunction with metabolic or renal factors, warranting further research. Interestingly, after additional adjustment for beta-blocker use, the association between 24 h and adverse outcomes re-emerged. Because beta-blockers directly lower heart rate and are commonly administered early after STEMI, failure to account for their use may partially obscure the relationship between intrinsic autonomic activation and adverse outcomes. Adjustment for beta-blockers may therefore reduce treatment-related confounding and better reveal the prognostic significance of persistently elevated 24 h. This aligns with previous evidence that pharmacological HR reduction improves prognosis in AMI, supporting the prognostic relevance of elevated HR rather than proving that active HR reduction itself improves outcomes [27].

We observed a linear increase in in-hospital adverse outcomes with an increase in AHR and 24 h. In multivariable analysis, AHR was better and more robust than 24 h in predicting the risk of in-hospital cardiac events. We observed that the probability of developing adverse in-hospital CV events was 3.3% and 18.6% for every 1 bpm and 5 bpm increment in AHR, respectively. Adverse in-hospital outcomes remained significant after adjusting for all the conventional risk factors and treatments. Thus, we have reconfirmed and highlighted the theory that elevated AHR is significantly associated with adverse in-hospital events in patients with STEMI.

Previous investigators have reported that elevated prehospital HR is independently associated with worse outcomes in STEMI [28]. However, we did not observe any association between FHR and in-hospital CV events in patients with STEMI. This discrepancy could be explained by our small sample size. Additionally, FHR was obtained during emergency evaluation, either in the ambulance or at the chest pain center, when patients’ physiological states are highly unstable. The variability in timing between symptom onset and hospital arrival could further obscure its prognostic accuracy. FHR likely reflects the acute ischemic burden and sympathetic activation rather than post-revascularization cardiac function recovery.

The observed associations between higher AHR or 24 h and adverse in-hospital events may be explained by several pathophysiological mechanisms. Following AMI, sympathetic overactivation leads to increased HR [29]. Elevated HR contributes to the pathogenesis of atherosclerosis by promoting oxidative stress, systemic inflammation, and endothelial dysfunction [30], all of which accelerate plaque formation. It is also linked to plaque rupture [31] and greater infarct size [32]. Moreover, increased HR enhances coronary thrombogenicity through elevated blood viscosity, platelet activation, and a procoagulant state [33]. Excessive HR elevates myocardial oxygen consumption [34], while reducing diastolic perfusion time, thereby exacerbating ischemia [35].

Experimental and clinical evidence supports the benefits of HR reduction. β-blockers attenuate myocardial ischemia and improve coronary perfusion and ventricular contractility [36], while ivabradine has been shown to reduce infarct size [37]. Contemporary ACS guidelines continue to support guideline-directed use of beta-blockers in appropriate patients, while also emphasizing individualized treatment according to hemodynamic status, left ventricular function, and contraindications. However, more recent randomized evidence has challenged the assumption that routine beta-blocker therapy uniformly improves outcomes in all post-MI patients, particularly those with preserved left ventricular ejection fraction. Therefore, although elevated AHR and 24 h in our study were associated with worse in-hospital outcomes, these observational findings should be interpreted as supporting the prognostic value of HR rather than proving that active HR reduction improves prognosis in this setting [38]. As previously noted by Parodi et al., it remains unknown whether heart-rate reduction itself results in improved outcomes in patients with acute myocardial infarction undergoing primary PCI [9]. In our study, even after adjusting for β-blocker use and other relevant medications, AHR and 24 h remained significantly associated with adverse in-hospital outcomes, supporting their independent prognostic value.

Further research is warranted to elucidate the prognostic value of high HR on the general population and patients with CVD. We, therefore, confirm and strengthen the theory that AHR and 24 h are significantly associated with adverse in-hospital events in patients with STEMI undergoing PPCI. Furthermore, AHR is a better and more reliable prognostic tool for determining adverse in-hospital outcomes. Consequently AHR may be an optimal clinical marker for the early identification of high-risk patients with STEMI.

This study has several limitations. First, this was a retrospective single-center observational study conducted in a relatively homogeneous Chinese population, which may limit external validity and generalizability to broader STEMI populations. Second, the final analytic cohort was reduced because patients with missing key HR measurements or incomplete clinical data were excluded, potentially introducing selection bias. In addition, patients with cardiogenic shock, atrial fibrillation, and other rhythm disturbances were excluded to ensure reliable HR assessment; therefore, the findings may not be generalizable to higher-risk patients with severe hemodynamic instability or arrhythmias. Third, although 99 patients experienced composite in-hospital adverse events, the endpoint was predominantly driven by acute heart failure, whereas the numbers of stroke and death events were relatively small. Consequently, the study was underpowered for reliable endpoint-specific multivariable analyses of these hard clinical outcomes. Fourth, no long-term follow-up data were available; therefore, the prognostic value of these HR measurements beyond hospitalization could not be evaluated. Fifth, detailed information regarding pre-admission medications, including chronic beta-blocker therapy and other rate-controlling agents, as well as the timing of in-hospital medication initiation and acute supportive interventions, was not consistently available. Some therapies may have been initiated in response to early clinical deterioration, potentially introducing treatment-related confounding and influencing HR measurements. Furthermore, detailed timing intervals, including symptom-to-contact and contact-to-admission times, were not consistently recorded and may also have affected HR assessment. Finally, given the limited number of outcome events relative to the number of covariates included in the fully adjusted models, overfitting cannot be excluded. In addition, some adjusted biomarkers and medications may represent intermediate biological pathways rather than pure confounders, which may complicate interpretation of the fully adjusted models. As with all observational studies, residual confounding cannot be completely excluded.

## Conclusion

In patients with STEMI undergoing primary PCI, elevated admission and 24-hour heart rates were associated with increased risk of adverse in-hospital events, predominantly acute heart failure, whereas first medical contact HR showed limited prognostic value. Among these measurements, admission HR demonstrated the most consistent association across multivariable models. However, given the retrospective observational design and limited numbers of hard clinical events, these findings should be considered hypothesis-generating and require validation in larger prospective studies.

## Data Availability

The data supporting the findings of this study are available upon reasonable request from the corresponding authors.

## Abbreviations

ACEI: Angiotensin-converting enzyme inhibitor
AHF: Acute heart failure
AHR: Admission heart rate
ALBu: Microalbuminuria
AMI: Acute myocardial infarction
ARB: Angiotensin II receptor blocker
BMI: Body mass index
CICU: Cardiac Intensive Care Unit
CK-MB: Creatine kinase-MB
CV: Cardiovascular
CVD: Cardiovascular disease
DBP: Diastolic blood pressure
DAPT: Dual antiplatelet therapy
ECG: Electrocardiogram
eGFR: Estimated glomerular filtration rate
EMR: Electronic medical record
FHR: First medical contact heart rate
FPG: Fasting plasma glucose
HF: Heart failure
HR: Heart rate
hs-CRP: High-sensitivity C-reactive protein
LVEF: Left ventricular ejection fraction
NT-proBNP: N-terminal pro-B-type natriuretic peptide
OR: Odds ratio
PCI: Percutaneous coronary intervention
PPCI: Primary percutaneous coronary intervention
STEMI: ST-segment elevation myocardial infarction
24HR: 24-hour heart rate
